# Molecular regulation of prostate cancer by Galectin-3 and estrogen receptor

**DOI:** 10.3389/fendo.2023.1124111

**Published:** 2023-03-03

**Authors:** Deborah Simão Souza, Carla Macheroni, Gustavo José Silva Pereira, Carolina Meloni Vicente, Catarina Segreti Porto

**Affiliations:** Laboratory of Experimental Endocrinology, Department of Pharmacology, Escola Paulista de Medicina (EPM), Universidade Federal de São Paulo (UNIFESP), São Paulo, SP, Brazil

**Keywords:** Galectin-3, β-catenin, ERα, ERβ, prostate cancer cells

## Abstract

Prostate cancer remains the most prevalent cancer among men worldwide. This cancer is hormone-dependent; therefore, androgen, estrogen, and their receptors play an important role in development and progression of this disease, and in emergence of the castration-resistant prostate cancer (CRPC). Galectins are a family of β-galactoside-binding proteins which are frequently altered (upregulated or downregulated) in a wide range of tumors, participating in different stages of tumor development and progression, but the molecular mechanisms which regulate its expression are still poorly understood. This review provides an overview of the current and emerging knowledge on Galectin-3 in cancer biology with focus on prostate cancer and the interplay with estrogen receptor (ER) signaling pathways, present in androgen-independent prostate cancer cells. We suggest a molecular mechanism where ER, Galectin-3 and β-catenin can modulate nuclear transcriptional events, such as, proliferation, migration, invasion, and anchorage-independent growth of androgen-independent prostate cancer cells. Despite a number of achievements in targeted therapy for prostate cancer, CRPC may eventually develop, therefore new effective drug targets need urgently to be found. Further understanding of the role of Galectin-3 and ER in prostate cancer will enhance our understanding of the molecular mechanisms of prostate cancer development and the future treatment of this disease.

## 1 Introduction

According to World Health Organization statistics, about 1.41 million new cases of prostate cancer were detected in 2020 (1). The androgen receptor (AR) is the main factor in the pathogenesis of this disease (reviewed by 2), and most patients benefit from androgen deprivation therapy, but disease recurrence and the emergence of castration-resistant prostate cancer (CRPC) are frequent after this treatment (reviewed by 3). Treatments for CRPC, that prolong the life of the patient, have emerged, including AR pathway inhibitors, radioisotope therapy, systemic taxane chemotherapy, and cell immunotherapy. However, such therapeutic strategies are limited (reviewed by 4, 5). Thus, the high prevalence of these tumors, lack of effective biomarkers and limited effective treatment highlight the importance of basic research in this disease for further treatment. This review provides an overview of the current and emerging knowledge on Galectin-3 in cancer biology with focus on prostate cancer and the interplay with estrogen receptor (ER) signaling pathways, present in androgen-independent prostate cancer cells. It is important to mention that the promoter region of the human LGALS3 gene contains regulatory elements for several transcription factors (6; reviewed by 7) and ERs may interact with these transcription factors (reviewed by 8). ERs have both classical transcriptional nuclear properties and membrane-initiated rapid action that may function either separately as distinct pathways, or together as a fully integrated network (reviewed by 9). These molecular mechanisms induced by activation of ERs may be involved in the expression of the GAL-3 in androgen-independent prostate cancer cells.

## 2 Structural characteristics, nucleus and cytoplasmic shuttling and functions of the Galectin-3 in cancer

Galectin proteins are characterized by specific binding of β-galactosides through evolutionarily conserved sequence elements of the carbohydrate-recognition domain (CRD). Galectin family consists of 15 members, divided into three main groups: (*i*) prototype group (Galectin-1, -2, -5, -7, -10, -11, -13, -14, and -15); (*ii*) tandem repeat group (Galectin-4, -6, -8, -9, and -12); (*iii*) and chimera type (Galectin-3) (reviewed by 7).

The human 29- to 35-kDa protein Galectin-3 is encoded by the *LGALS3* gene (10) and this protein has three distinct structural motifs: (*i*) a short N-terminal domain containing a serine phosphorylation site; (*ii*) a repetitive proline-rich collagen-α-like sequence cleavable by matrix metalloproteases; and (*iii*) a globular C-terminal domain containing a carbohydrate-binding motif and an NWGR anti-death motif (reviewed by 7, 11).

The expression of the Galectin-3 is observed mainly in the cytoplasm, and also in the nucleus. This protein can be secreted by non-classical secretory pathways, by a mechanism that remains unclear, but a study has shown the involvement of exosomes (12). In addition, both the nucleus and cytoplasmic shuttling of the Galectin-3 has been reported (13, 14). The N-terminal domain contains a serine 6 phosphorylation site, which plays an important role in nuclear transport (reviewed by 7, 15, 16). The CRD region is important for localization of the Galectin-3 in cells, since both nuclear import sequences (NLS) and nuclear export sequences (NES) are present in this region (reviewed by 7, 14, 17). Galectin-3 is translocated to the nucleus through the importin-α/β, and the amino acid residue Arg (224) is essential for its active nuclear translocation and its molecular stability (15, 16). Galectin-3 carries a functional NES, recognized by the CRM1 exportin, being inhibited by Leptomycin B (18). The secreted Galectin-3 mediates cell migration, through the binding with galactose-containing glycoproteins in the cell surface. Cytoplasmic Galectin-3 exhibits anti-apoptotic activity and regulates several signal transduction pathways, whereas nuclear Galectin-3 has been associated with pre-mRNA splicing and gene expression (reviewed by 17, 19).

Galectin-3 is involved in many significant biological processes linked to cancer development and progression (20; reviewed by 21, 22; 19, 23). In addition, Galectin-3 exerts a role as a pro-tumor factor by acting within the tumor microenvironment to suppress immune surveillance (24).

The increase in the expression of the Galectin-3 is observed by RT-PCR or immunohistochemistry in different types of cancers, including breast, colon, gastric, hepatocellular, anaplastic large-cell lymphoma, head and neck squamous cell, tongue, non-small cell lung, and well differentiated thyroid (reviewed by 23, 25). Other studies showed a decrease in the expression of the Galectin-3 in breast, ovary, prostate cancers, advanced uterine adenocarcinoma, basal cell of the skin, epithelial skin, and malignant salivary gland neoplasms, compared to the corresponding normal tissue (reviewed by 23, 26).

## 3 Molecular regulatory mechanisms responsible for the expression of the Galectin-3 in cancer

Changes in the expression of the Galectin-3 are commonly seen in cancer and pre-cancerous conditions. However, the molecular regulatory mechanisms responsible for the level of expression of the Galectin-3 in tumor cells are not yet clear. Understanding these molecular mechanisms could contribute to the development of new approaches for cancer treatment.

DNA methylation in the gene promoter region is not the only factor regulating the expression of the Galectin-3 (27), since several features are involved in the protein regulation, in both transcriptional and translational levels (reviewed by 7). The levels of the Galectin-3 are not directly increased by a specific factor, but it would be dependent on the differentiation status of the cells or the type of the tissues.

Several features, for exemple runt-related protein (RUNX) family, homeodomain-interacting protein kinase 2 (HIPK2), nuclear factor kB (NF-kB), inflammation cytokines, and some intracellular signal pathways are involved in the regulation of the expression of the Galectin-3 and on cell growth, differentiation, proliferation, apoptosis, migration, angiogenesis, invasion, metastasis, and radiation resistance (reviewed by 7, 23).

Regarding these factors, two binding sites for RUNX1 and one binding site for RUNX2 were identified in the *LGALS3* promoter region in human pituitary cell line HP75. Knockdown of either RUNX1 or RUNX2 resulted in downregulation of the Galectin-3, decreasing cell proliferation (28). HIPK2 is important for repression of the Galectin-3 upon induction of p53-dependent apoptosis (29).

NF-κB and Jun induced the expression of the Galectin-3 by UV-light in glioblastoma cells (30). Another study showed that Nucling, which is a stress-inducible protein associated with apoptosomes, inhibits the expression of the Galectin-3 by interfering with activation of the NF-kB (31).

Ras/mitogen-activated protein kinase (MAPK) kinase 1 (MEK1 or MKK1)- dependent/protein-1 activator (AP-1) signal transduction pathway plays an important role in the expression of the Galectin-3 in macrophages stimulated by Phorbol 12-myristate 13-acetate (PMA) (32).

Taken together, it is evident that the regulation of expression of the Galectin-3 is a complex, fine-tuned mechanism that involves numerous transcription factors and signaling pathways, and which depends on cell type, external stimuli and environmental conditions.

## 4 Expression of the Galectin-3 and prostate cancer

The studies in prostate cancer have mainly focused on Galectin-1 and Galectin-3, but the importance of Galectin-4, Galectin-7, Galectin-8, and Galectin-9 has also been highlighted (reviewed by 11, 33, 34). In relation to Galectin-3, different studies have indicated decrease of the expression over disease progression. In primary prostate cancer and metastatic disease, the expression of the Galectin-3 decreased compared to normal and premalignant tissue (35, 36). Normal prostate tissue showed heterogenous expression of the Galectin-3. In stage II tumors, however, a dramatic decrease in the expression of the Galectin-3 in both PIN and tumor sections was detected, with only 10.5% of these samples expressing this protein (37). In prostate cancer and adjacent non-tumoral tissue, the expression of the Galectin-3 was also examined by van den Brûle et al. (38). They found that most of the non-tumoral tissue exhibited moderate immunostaining for Galectin-3 localized in both nucleus and cytoplasm and prostate cancer cells showed decrease of the expression of the Galectin-3 or not expression compared to the normal tissue. In the hormone-sensitive prostate cancer tissue when compared to the respective benign tissue either localized far distant from the malignant lesion or directly neighboring the primary tumor, the expression of the Galectin-3 was significantly decreased in the prostate cancer tissue (39). The cellular localization of the Galectin-3 was shown in benign, adjacent-benign and tumor tissues. Median Galectin-3 staining scores significantly decreased from benign to adjacent-benign and to tumor tissues (40).

In addition, the expression of the Galectin-3 was also investigated in normal, BPH, and various stages of the prostate cancer which showed decreasing immunopositivity during stage evolution. Galectin-3 was found strongly expressed both in nucleus and cytoplasm in normal, BPH, and HGPIN, a precursor lesion to development of invasive prostatic adenocarcinoma tissues. Moreover, localization of the Galectin-3 seems to vary during tumor stage evolution. In particular, stage I tumors showed a strong immunopositivity in both the nucleus and the cytoplasm, whereas in more advanced stages, immunostaining was less intense and localized mainly in cytoplasm, with rare, occasional nucleus positivity (41).

On the other hand, recent study using biopsy samples, representing different stages of the primary prostate cancer, showed that prostate specific membrane antigen (PSMA), Galectin-1 and Galectin-3 are the most abundantly expressed glycoproteins. Galectin-3 correlated with the expression of PSMA, independently of PSA and Gleason score at diagnosis (42). The level of the Galectin-3 in the serum increased in metastatic prostate cancer (43) or decreased in prostatic adenocarcinomas (44) compared to healthy individuals. A prospective clinical study analyzed Galectin-3 and prostate specific antigen (PSA) (and their respective autoantibodies) levels in the serum and described positive associations between Galectin-3 and PSA levels in 76 men with different stages of prostate cancer and in 19 healthy control individuals (45, 46).

Galectin-3, in addition to different oligomeric forms, can to be cleaved by matrix metalloproteinases (MMP)-2/-9. In mouse models of breast and prostate cancers, this cleavage is associated with angiogenesis, tumor growth, and resistance to apoptosis (47).

Emerging studies are associating the expression of the Galectin-3 with the immune response against prostate cancer, as well as to the success of effective immunotherapy. Tiraboschi et al. (48) showed that when Galectin-3 is expressed by prostate tumor cells, it can control the tumor growth and decrease the number of tumor infiltrated T cells, suggesting that this protein is the principal immunological checkpoint responsible of the failure of immunotherapy in advanced prostate cancer. In addition, low doses of docetaxel inhibited the expression of the Galectin-3 in prostate cancer cells as well as in clinical samples of patients with metastatic cancer and CRPC, controlling tumor recurrence by increasing proliferation and infiltration of CD8+ cytotoxic T (49).

Further investigation is important to elucidate the relationship between the expression of the Galectin-3 and its regulation, cleavage and function in different stages of prostate cancer and CRPC.

Our laboratory and other groups have shown the expression of the Galectin-3 in androgen-independent prostate cancer cells PC-3 (derived from bone metastasis) and DU-145 (derived from brain metastasis), used *in vitro* and in xenograft implants, as a CRPC models (50–54). The expression of the Galectin-3 is higher in DU-145 cells and human post pubertal prostate epithelial cells (PNT1A) than in PC-3 cells (54). On the other hand, the androgen-dependent prostate cancer cell LNCaP do not express Galectin-3 (51, 52). Furthermore, neither overexpression of the Galectin-3 in LNCaP cells (LNCaP-GAL-3 cells) (51, 52) nor knockdown of the Galectin-3 in PC-3 cells change the expression of AR (51); similarly, overexpression of the AR in PC-3 cells does not have regulatory effect on the expression of the Galectin-3 (51). Taken together, these studies indicated that AR and Galectin-3 are not involved in the regulation of each other’s protein expression. Other hormones and their receptors, such as ER, and growth factors present in tumor microenvironment may be involved in the regulation of the expression of the Galectin-3.

LNCaP-GAL-3 cells promote both cell migration and invasion in an androgen-independent manner compared to control LNCaP cells and the transcriptional activity of the AR with treatment with dihydrotestosterone is enhanced in these cells (52). Furthermore, several AR-target genes, such as kallikrein related peptidase 3 (KLK3), and transmembrane protease, serine 2 (TMPRSS2) are increased (52). These AR-target genes in LNCaP-GAL-3 are not fully inhibited by anti-androgen, such as bicalutamide or MDV3100, whereas their expression in LNCaP cells is completely inhibited by anti-androgen, suggesting that Galectin-3 may be involved in resistance to anti-androgen (52). Galectin-3 also enhances anchorage-independent growth and xenograft tumor growth even after castration (52).

Knockdown of the Galectin-3 by siRNA reduced cell migration, invasion, cell proliferation, anchorage-independent colony formation of the PC-3 cells (50), and impaired tumor growth (50, 52). Inhibition of the Galectin-3 with pharmacological strategies impaired angiogenesis and metastasis (55; reviewed by 11). Furthermore, Galectin-3 can inhibit apoptosis in prostate cancer cells (51, 56).

Taken together, these results demonstrated that the levels and the cellular location of the Galectin-3 vary during prostate cancer progession, since the malignant transformation of prostate cells is associated with cellular redistribution of the Galectin-3 and a decrease in tissue levels of this protein (57). Functional studies using prostate cancer cell lines suggest that the expression of the Galectin-3 is not regulated by AR, but other hormones and their receptors, such as ER, could be involved in the regulation of the expression of the Galectin-3.

## 5 Molecular regulatory mechanisms responsible for the expression of the Galectin-3 induced by estrogen signaling in androgen-independent prostate cancer cells

17β-estradiol (E2) impacts normal and malignant tissue development *via* estrogen receptors ERα (ESR1) and ERβ (ESR2), either through ligand-activated transcriptional regulation (genomic pathway) or by triggering cytoplasmic-signaling cascades (rapid action or nongenomic pathway). The possible convergence of genomic and rapid pathways on target genes is an attractive mechanism by which ER can finely regulate the gene expression in different cells (reviewed by 9, 58).

ER, in addition to AR, plays an important biological function as a transcription factor and regulatory protein in prostate cancer (reviewed by 59–62). The traditional paradigm regarding the roles of the two ERs in the prostate is that ERα is oncogenic and promotes cell proliferation and survival, whereas ERβ is predominantly protective, being anti-carcinogenic and pro-apoptotic (reviewed by 59, 62). However, increasing evidences have shown that ERβ may be potentially oncogenic in prostate cancer (reviewed by 59). A variety of factors contribute to the uncertainties surrounding molecular action and tissue expression of ERs. Some antibodies used for ERβ are not specific and/or inadequately validated; the immunohistochemical analyses of estrogen receptors rely on cell permeabilization and reagent specificity, as well as changes in results have been shown depending on tissue fixation and processing, including antigen retrieval methods (reviewed by 63–65). The presence of ERβ splice variants in human tissues and cell lines, that have modified C-terminal and will not be recognized by antibodies raised against the C-terminal peptide of ERβ. Therefore, antibodies raised against the N-terminal of ERβ are important because they evaluate ERβ1 and all of its splice variants, but it is important that the epitopes should not include threonine, serine or tyrosine residues which are phosphorylated (reviewed by 65). A further problem is the passage of the acquired cell line, its time in culture and the presence of Mycoplasma. All cell lines need to be authenticated by DNA profiling, and contamination by Mycoplasma and other microorganisms excluded (66).

Our group has shown the presence of ERα and ERβ by Western blot and immunofluorescence analyses in androgen-independent prostate cancer cells PC-3 and DU-145 and in human post pubertal prostate epithelial cells PNT1A, using different antibodies for ERα and ERβ (67, 68), previously validated (64) and also positive control cells for these receptors (67, 69). On the other hand, ERβ was not observed in PC-3 and DU-145 cells (70) and also in human prostate (71). One of the problems in these studies may be the use of N-terminal antibodies, as previously reported (reviewed by 65) and/or different sensitivity of antibodies used depending on the expression level of ERβ, as shown in male reproductive organs (reviewed by 72). Furthermore, immortalized cell lines may have variable expression of certain factors across passage numbers and laboratories (66). We suggest that the data must be interpreted carefully in relation to the expression of ERβ and immunohistochemical and Western blot studies need to be supplemented with other methods.

**Figure 1:**
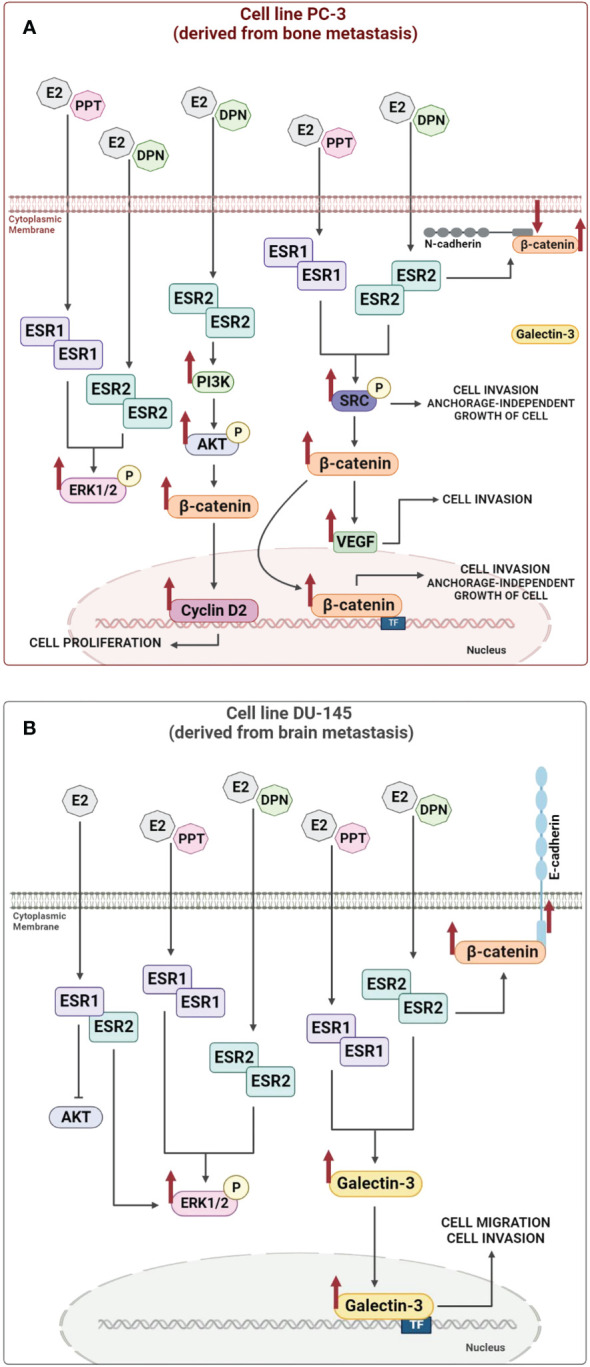
Illustration of the molecular regulatory mechanisms responsible for the expression of the non-phosphorylated β-catenin and Galectin-3 induced by estrogen receptor activation. Activation of estrogen receptors ESR1 and ESR2 by E2, PPT or DPN increases (arrows) the phosphorylation of SRC, ERK1/2 or AKT and the expression of non-phosphorylated β-catenin, Cyclin D2, VEGF and Galectin-3, which in turn can modulate nuclear transcriptional events, such as, proliferation, migration, invasion, and anchorage-independent growth of androgen-independent prostate cancer cells PC-3 (**A**) and DU-145 (**B**) (see 54, 67, 68, 73–76 for more information). TF (transcription factor).

In androgen-independent prostate cancer cells PC-3 and DU-145, ERα and ERβ are mostly located outside the cell nucleus (67, 68), while in human post pubertal prostate epithelial cells PNT1A (68) and in positive control cells (primary Sertoli cells and human testicular embryonal carcinoma NT2/D1 cells; 67, 69), these receptors are predominantly located in the nucleus, using these same antibody. The activation of ERα and ERβ increases the phosphorylation of extracellular signal-regulated kinase1 and 2 (ERK1/2) in PC-3 and DU-145 cells (67, 68) (illustration in **Figures 1A, B**) and non-receptor tyrosine kinase (SRC) in PC-3 cells (76) (illustration in **Figure 1A**). Furthermore, activation of ERβ in PC-3 cells increases the phosphorylation of serine/threonine kinases (AKT) (73). The activation of the complex ERα/SRC or ERβ/SRC increases the expression of the active non-phosphorylated β-catenin and vascular endothelial growth factor (VEGF) (75) (illustration in **Figure 1A**). Furthermore, the activation of ERβ increases the migration, invasion, and anchorage-independent growth of PC-3 cells (75). The activation of ERα also increases the invasion and anchorage-independent growth of these cells (75, 77) (illustration in **Figure 1A**). These effects are blocked by pretreatment with PKF 118–310, a compound that disrupts the complex β-catenin/TCF/LEF, suggesting that ERs/β-catenin are involved in all cellular characteristics of tumor development *in vitro* (75). All together, these results support an oncogenic role for ERα and ERβ in PC-3 cells.

In DU-145 cells, in addition to the presence of ERα and ERβ, formation of ERα/β heterodimers is observed and the activation of ERK1/2, but not AKT, by these receptors (68) (illustration in **Figure 1B**). The treatment with E2, ERα-selective agonist PPT or ERβ-selective agonist DPN for 24h increases the expression of the Galectin-3 compared to untreated DU-145 cells (control) (54) (illustration in **Figure 1B**). The activation of ERβ also increases the expression of β-catenin in the cellular membrane of DU-145 cells (74) (illustration in **Figure 1B**).

It is important to mention that the promoter region of the human *LGALS3* gene contains binding sites for specificity protein 1 (Sp1), cAMP response element binding protein (CREB), AP-1, NF-κB and sis-inducible element (SIE) (6; reviewed by 7) and several of these transcription factors interact with ERs (reviewed by 8) and may play a role on the expression of the Galectin-3. ERs also activate two major pathways regulating cell proliferation and survival, SRC/MAPK and PI3K/AKT pathways (rapid or non-genomic signaling) (reviewed by 9, 78). Indeed, in DU-145 cells, the activation of ERα and ERβ increases the phosphorylation of ERK1/2, but not of AKT (68). Thus, direct activation of signaling cascades (non-genomic activity) by ERs combined with the transcriptional regulation (genomic activity) may be involved in the expression of the Galectin-3 in DU-145 cells. The direct transcriptional regulation remains to be explored in these cells. Furthermore, the activation of ERα and ERβ increases the migration and invasion of the DU-145 cells. These processes are inhibited by VA03 (specific inhibitor of Galectin-3), indicating the involvement of the complex ERα- ERβ/Galectin-3 (54).

In summary, ERs can mediate the rapid E2 actions in PC-3 (illustration in **Figure 1A**) and DU-145 cells (illustration in **Figure 1B**) and, respectively, increase the expression of non-phosphorylated β-catenin and Galectin-3, which in turn can modulate nuclear transcriptional events, such as, proliferation, migration, invasion, and anchorage-independent growth of these cells (illustration in **Figure 1**), indicating the oncogenic role of ERα and ERβ in both cells.

The activation of ERβ by selective-agonist DPN also promoted survival and migration of the CPEC cell line (cells expressing prostate-specific antigens), established from prostate cancer patients (79). In LNCaP cells (androgen-dependent prostate cancer cell), ERβ was able to drive the cells into S-phase and promote cell proliferation and epidermal growth factor secretion (80). In CRPC, ERβ plays a role in AR-dependent gene transcription (81), mediating the transition from hormone-sensitive to CRPC (82). The expression of ERβ is augmented in bone and lymph node metastases (83) and high expression of ER correlates with poor clinical prognosis (82, 84).

## 6 Conclusions and future perspectives

This review highlights the importance of Galectin-3 in the pathogenesis, diagnosis and treatment of prostate cancer. The activation of signaling cascades by membrane/cytoplasm-localized ERs is involved in the expression of Galectin-3 and non-phosphorylated β-catenin in androgen-independent prostate cancer cells. Nuclear ERs collaboration in this process still remains to be explored. ER, Galectin-3 and non-phosphorylated β-catenin can modulate nuclear transcriptional events, such as, proliferation, migration, invasion, and anchorage-independent growth of these cells.

It is important to mention that previous studies have shown that Galectin-3 binds to β-catenin/TCF complex, colocalizes in the nucleus, and induces the transcriptional activity of TCF-4. The β-catenin-Galectin-3-binding sequences were identified in the N-and C-termini of the proteins encompassing amino acid residues 1 to 131 and 143 to 250, respectively (85). In human colon cancer cells, Galectin-3 mediates AKT phosphorylation, thereby increasing phosphorylation of Glycogen synthase kinase-3β (GSK-3β) and decreases its activity and reduction in β-catenin degradation (86). β-Catenin can then translocate to the nucleus, bind to TCF4, and activate the transcription of its specific target genes (86). Thus, the presence of ER, Galectin-3 and non-phosphorylated β-catenin in androgen-independent prostate cancer cells shows a complex picture, which remains to be explored.

Several therapeutic avenues are emerging from the characterization of these signaling pathways discussed in this review. Investigators have made great progress in understanding how prostate cancer can disseminate early in the disease course and relapse months or decades later. Although clinical researchers have had modest gains thus far, much more work is needed to refine the basic science and translate this knowledge to the clinic.

## Ethics statement

All experimental procedures were approved by the Research Ethical Committee at EPM-UNIFESP (#3527220917).

## Author contributions

DSS: Conceived and designed the analysis; Collected the data; Performed the analysis; Wrote the manuscript. CM: Contributed with analysis tools. CMV: Contributed data or analysis tools. GJSP: Conceived and designed the analysis, performed the revision of the manuscript. CSP: Conceived and designed the analysis, performed the revision of the manuscript. All authors contributed to the article and approved the submitted version.
